# Complete Genome Sequences of Two *Rhizobium* Strains Producing Azol(in)e-Modified Antibiotics

**DOI:** 10.1128/mra.00722-22

**Published:** 2022-10-13

**Authors:** Dmitrii Y. Travin, Dmitry Sutormin, Peter Mergaert, Konstantin Severinov

**Affiliations:** a Center of Life Sciences, Skolkovo Institute of Science and Technology, Moscow, Russia; b Institute of Gene Biology, Russian Academy of Science, Moscow, Russia; c Université Paris-Saclay, CEA, CNRS, Institute for Integrative Biology of the Cell (I2BC), Gif-sur-Yvette, France; d Waksman Institute for Microbiology, Rutgers, Piscataway, NJ, USA; University of Arizona

## Abstract

Rhizobia are known for their ability to establish symbiotic relationships with plants. The specialized metabolism of these bacteria remains understudied. Here, we report whole-genome sequences of two rhizobia producing narrow-spectrum antirhizobial azol(in)e-modified peptides: that of *Rhizobium* sp. Pop5, a phazolicin producer, and another of Rhizobium anhuiense T24, a trifolitoxin producer.

## ANNOUNCEMENT

Symbiotic nitrogen-fixing rhizobia compete for the host plants’ nodulation using a variety of mechanisms, including the production of antimicrobial compounds (1). Phazolicin (2) and trifolitoxin (3) are antirhizobial azol(in)e-containing antibiotics belonging to ribosomally synthesized and posttranslationally modified peptides (RiPPs). The genome of the trifolitoxin producer, isolated from the nodules of *Trifolium dubium* and originally classified as Rhizobium trifolii T24 (4), was not sequenced to date except for two small, 7.1- and 7.3-kbp regions (5, 6). The genome of phazolicin-producing *Rhizobium* sp. Pop5, isolated from the nodules of Phaseolus vulgaris, was available in contigs only (GCA_000295895.1).

*Rhizobium* sp. Pop5 was provided by Esperanza Martinez-Romero, and R. anhuiense T24 was obtained from USDA-ARS Culture Collection (USDA2124). Both strains were maintained on YM medium. Genomic DNA was extracted from overnight cultures grown at 28°C using GeneJET Genomic DNA purification kit (ThermoFisher). Pop5 DNA was sequenced using Oxford Nanopore Technologies (ONT) sequencing, while a combination of ONT and Illumina sequencing was performed for T24 DNA. For Nanopore sequencing, libraries were prepared with nonsheared DNA using the Ligation Sequencing kit 1D (SQK-LSK109; ONT) and EXP-NBD104 native barcoding expansion according to standard protocols. Genomic DNA was subjected to End Repair and A tailing by NEBNext FFPE DNA Repair mix and NEBNext Ultra II End repair/dA-tailing module (NEB). Sequencing adaptors were ligated using NEBNext Ultra II DNA Ligation module (NEB) after AMPure XP (Beckman Coulter) purification. The final product was cleaned using LFB buffer. MinION sequencing was performed using R9.4 flow cell (FLO-MIN106D; ONT). Base calling was performed using Guppy 6.0.1 (7) with the “sup” mode. For Illumina sequencing, DNA was sheared using Covaris sonicator, and 200- to 400-bp fragments were size selected with AMPure XP beads. The library was prepared using NEBNext Ultra II DNA Library Prep kit (NEB). Sequencing was performed on MiSeq with the 250 + 250-bp paired-end protocol.

Illumina reads were filtered and trimmed with Trimmomatic v 0.39 (8). For T24, a draft genome was assembled with Unicycler (v 0.5.0) (9) using ONT reads. The assembly was subsequently polished with medaka (v 1.5.0), Polypolish (v 0.5.0) (10), and POLCA (v 4.0.8) (11) using ONT and Illumina reads. For Pop5, a draft genome was assembled with Unicycler (v 0.5.0), and the assembly was polished with medaka (v 1.5.0). Default parameters were used for all software unless otherwise specified. Replicons were circulated and rotated automatically using Unicycler and annotated using PGAP 6.1.

**FIG 1:**
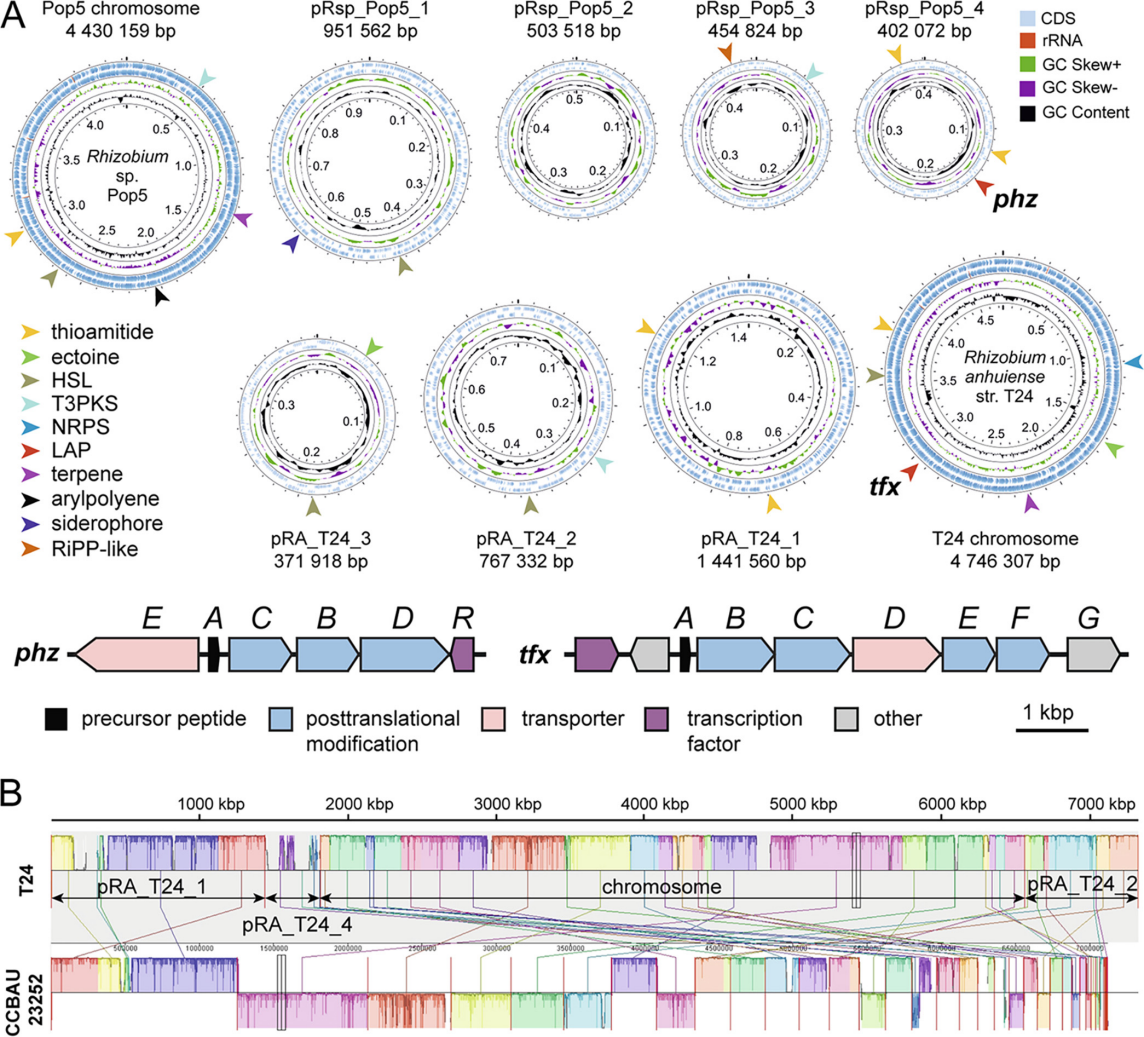
(A) Genome organization of the two sequenced *Rhizobium* strains visualized using Proksee (https://proksee.ca/). The chromosomes and plasmids are shown not to scale. Colored arrowheads indicate the locations of biosynthetic gene clusters identified by antiSMASH. Schematic representation of *phz* and *tfx* clusters is shown below with the functions of encoded proteins listed. CDS, coding sequence; HSL, homoserine lactone; T3PKS, type III polyketide synthase; NRPS, nonribosomal peptide synthetase; LAP, linear azol(in)e-containing peptide; RiPP, ribosomally synthesized posttranslationally modified peptide. (B) Alignment of the whole-genome of *R. anhuiense* T24 and the draft genome of *R. anhuiense* CCBAU 23252^T^ (GCA_003985145.1) (14) generated with Mauve (15).

Both genomes contain multiple replicons, including the chromosome and several plasmids (Table 1). An antiSMASH (12) search revealed 12 biosynthetic gene clusters per genome (Fig. 1A). The trifolitoxin *tfxABCDEFG* cluster is located on the chromosome, while the phazolicin cluster *phzEACBDR* is encoded on the smallest plasmid (Fig. 1A). According to average nucleotide identity (ANI) analysis by JSpeciesWS (13), the genome of R. leguminosarum T24 is 99.08% identical (89.78% aligned nucleotides) to that of *R. anhuiense* CCBAU 23252^T^ (GCA_003985145.1) (14) (Fig. 1B). Accordingly, the strain is reclassified as *R. anhuiense* bv. *trifolii* T24.

**TABLE 1:** Accession numbers, assembly, and run statistics for the two *Rhizobium* genomes

	*Rhizobium* sp. Pop5	Rhizobium anhuiense T24
BioSample accession no.	SAMN28987821	SAMN28987535
BioProject accession no.	PRJNA848232	PRJNA848223
GenBank accession no.	CP099398 CP099399 CP099400 CP099401 CP099402	CP098801 CP098802 CP098803 CP098804
Illumina sequencing reads		SRR20001171
ONT sequencing reads	SRR20000763	SRR20001172
Total reads length (Illumina) (bp)		463,844,988
Total reads length (ONT) (bp)	338,208,140	185,875,667
No. of reads (Illumina)		923,994
No. of reads (ONT)	41,698	25,553
*N*_50_ (ONT) (bp)	16,641	13,212
Genome size (bp)	6,742,135	7,327,117
GC content (%)	60.93	60.97
No. of replicons	5	4
Replicon sizes (bp)	4,430,159 951,562 503,518 454,824 402,072	4,746,307 1,441,560 767,332 371,918

### Data availability.

The genome sequences reported were deposited in DDBJ/ENA/GenBank databases; the raw reads were deposited in the NCBI Sequence Read Archive. Accession numbers are provided in Table 1.
